# Male-biased sex ratio in the crawling individuals of an invasive naticid snail during summer: implications for population management

**DOI:** 10.1038/s41598-022-12144-1

**Published:** 2022-05-12

**Authors:** Kazuki Yoshida, Tomoka Setogawa, Toshiyuki Sato, Manabu Yamada, Tatsuma Sato, Kaoru Narita, Akira Matsumoto, Takeshi Tomiyama

**Affiliations:** 1 Graduate School of Integrated Sciences for Life, Hiroshima University, Higashi-Hiroshima, Hiroshima 739-8528 Japan; 2 Fukushima Prefectural Research Institute of Fisheries Resources, Soma, Fukushima 976-0005 Japan

**Keywords:** Behavioural ecology, Invasive species

## Abstract

The naticid snail *Laguncula pulchella* is an invasive species that preys on clams in tidal flats and has serious impacts on clam fisheries in Japan. *Laguncula pulchella* burrow in sand, but often crawl on sediment surfaces during low tide. We investigated seasonal changes in the abundance and sex ratio of crawling *L. pulchella* during the daytime at Matsukawaura Lagoon, Japan, from March to October from 2015 to 2019. The density of crawling individuals peaked in July. The sex ratio of crawling individuals varied with months and years but was significantly biased towards males during the main copulation period (July–August); males accounted for 77–98% of the mature crawling individuals (≥ 25 mm shell height). The somatic condition of mature males declined from June to August, whereas that of females was constant during this period. These results indicate that mature males actively come to the sand surface during low tide to search for females for copulation from July to August. Fishermen make efforts to remove crawling individuals in summer, but the male-biased sex ratio must also be considered for effective population control of this species.

## Introduction

Invasive species are a threat to biodiversity, and their control is an important issue in ecosystem conservation^1–3^. To reduce the impact of invasive species on native species, eradication of the alien species is ideal but is difficult for marine organisms^4^. Detailed biological knowledge of the target species is essential for efficient and effective eradication, even though successful cases of invasive species management were not necessarily attributed to the population biological research^5^.

Naticid gastropods (moonsnails) are top predators of tidal flat ecosystems that prey upon clams and other gastropods^6–8^. Their behavioral patterns are important topics in the conservation of clams and the management of tidal flat ecosystems. Naticids burrow in the sand and some species crawl on the sand surface, thereby leaving trails that enable humans to identify the locations of burrowing individuals^9,10^. The crawling behavior of naticids on the sediment surface is known to be related to foraging. For example, *Natica unifasciata* crawls on mud flats for foraging at low tide^11,12^. Savazzi and Reyment^10^ associated the crawling behavior of naticids in an exposed sandy flat, with prey capture and defined it as "subaerial hunting". This behavior has also been observed in *Notocochlis gualteriana* (Savazzi and Reyment^10^) and *Paratectonatica tigrina*^13^. However, detailed information, such as the seasonal frequency of crawling behavior and ecological significance of that behavior, is still lacking for naticids.

**Figure 1:**
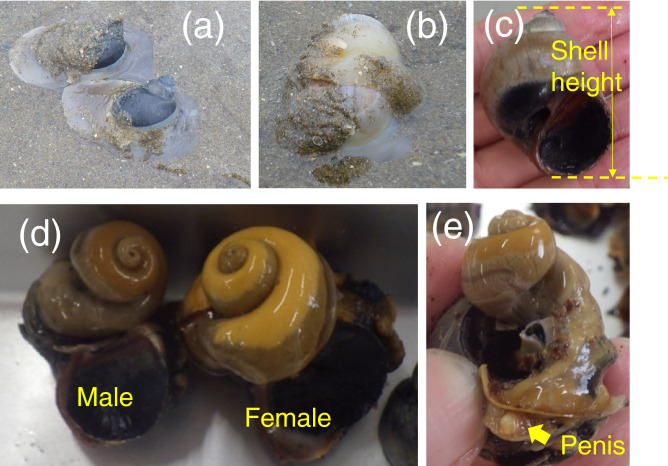
Photographs of *Laguncula pulchella*. (**a**) Crawling behavior of two individuals. (**b**) Shell mounting behavior. (**c**) Shell height (SH). (**d**) Soft body of a male and female after removing large part of the shell. (**e**) Male individual having a penis.

The naticid *Laguncula pulchella* (formerly described as *Euspira fortunei*) is an invasive species in northern Japan. This species was introduced to Japan in the late 1990s^14^ when it was unintentionally mixed with the imported asari (Manila) clam *Ruditapes philippinarum* and released into tidal flats. In Matsukawaura Lagoon, northern Japan, *L. pulchella* was first found in 2002 and thereafter its population rapidly increased and remained at a high level since 2004^15^. Its predation impacts on the asari clam and other bivalves have been of serious concern^14,16–20^. *Laguncula pulchella* bury themselves in sediments and appear on tidal flats at low tide^17^; they crawl on the sand surface (Fig. 1a) for ~ 30 min and thereafter bury themselves in the sand, leaving prominent trails. Based on this habit, the extermination of crawling individuals has been carried out by fishermen and local people. Fishermen have made efforts to manually remove the crawling individuals and egg collars of *L. pulchella* in Matsukawaura Lagoon during summer and autumn, respectively, since 2004. However, the efforts did not lead to a marked decline in the *L. pulchella* population^15,21^, because many individuals remained buried. Biological significance of the crawling of *L. pulchella* on the sediment surface should be understood for the efficient extermination. They have the size preference of prey clams^22,23^, and therefore crawling is effective to search prey in a wide area, although *L. pulchella* can capture prey in the substratum and bore drillholes on the clam shells^24^. Nonetheless, it is still unclear whether *L. pulchella* come to the sand surface only for hunting.

In northern Japan, *L. pulchella* copulates mainly in July and August, with a male mounting on top of a female (shell mounting; Fig. 1b)^17^. The sexes are fixed in naticids^25^. The initial maturation of females occurs from 2 years of age at a shell height of ≥ 25 mm^17^. Females can store sperm^17^ and lay egg collars between September and October^26^. Juveniles hatch from egg collars 30–40 days after egg laying^27^. Fishermen have continued removing *L. pulchella* to reduce predation on asari clams, but a population decline in *L. pulchella* has not been observed^15^. To achieve better management of this predatory gastropod and conservation of prey clams, the frequency and ecological importance of the crawling behavior of *L. pulchella* should be understood.

This study aimed to examine seasonal changes in the abundance of crawling individuals of an infaunal naticid, *L. pulchella*, on tidal flats in Matsukawaura Lagoon, northern Japan. Specifically, we focused on the abundance and sex ratio of crawling individuals because crawling behavior may vary between males and females or between seasons. We tested the null hypothesis that the sex ratio of crawling individuals was consistently 1:1 between males and females, irrespective of season. Furthermore, the shell size and somatic condition of crawling individuals were investigated to elucidate sexual differences in biological characteristics.

## Results

### Density and shell size of the crawling *L. pulchella* individuals

**Figure 2:**
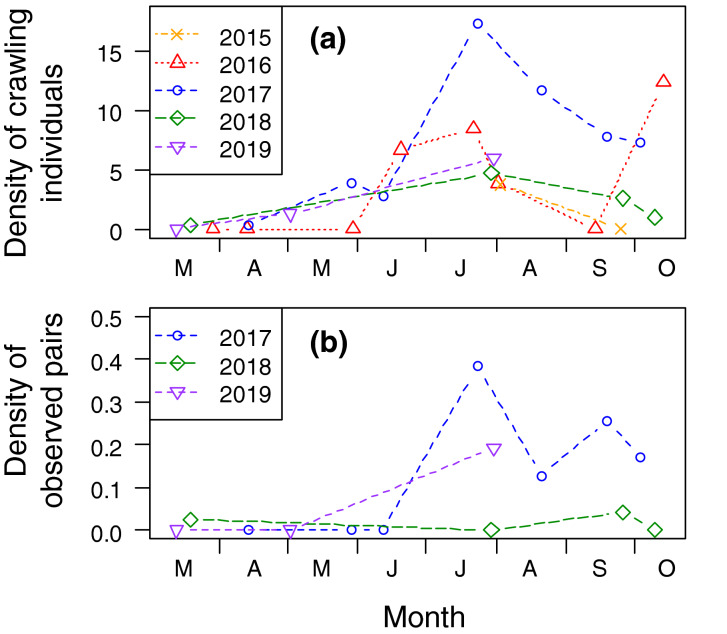
Seasonal changes in densities (the number per 100 m^2^) of (**a**) crawling *Laguncula pulchella* individuals and (**b**) mounting pairs found in the field.

The density of crawling *L. pulchella* individuals was high (2–17 individuals/100 m^2^) from June to October (Fig. 2a). The highest density was observed in July, except in 2016. Pairs that exhibited shell mounting were not observed between April and June, whereas the highest number of pairs was observed in July (Fig. 2b).

**Figure 3:**
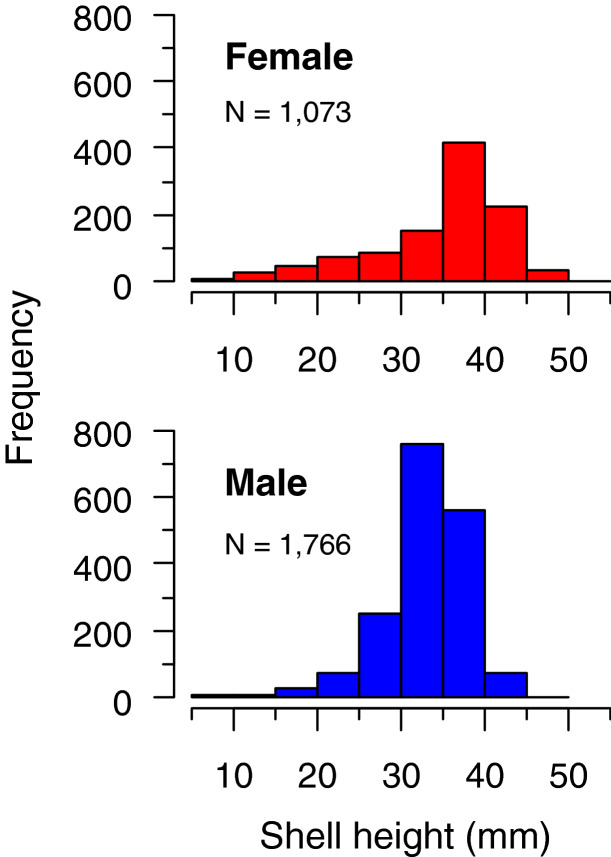
Shell height composition of crawling *Laguncula pulchella* individuals collected in Matsukawaura Lagoon between 2015 and 2019.

The shell height (SH, mm; Fig. 1c) of crawling *L. pulchella* was significantly larger in females (7–51 mm, median = 37.0 mm; n = 1073) than in males (8–49 mm, median = 33.6 mm; n = 1766; Mann–Whitney *U* test, *U* = 1,228,774, *P* < 0.001). The proportion of individuals ≥ 35 mm was greater in females (63.5%) than males (36.0%, Fig. 3), and the frequency distribution was significantly different between the sexes (Kolmogorov–Smirnov test, *D* = 0.34, *P* < 0.001).

### Sex ratio

**Figure 4:**
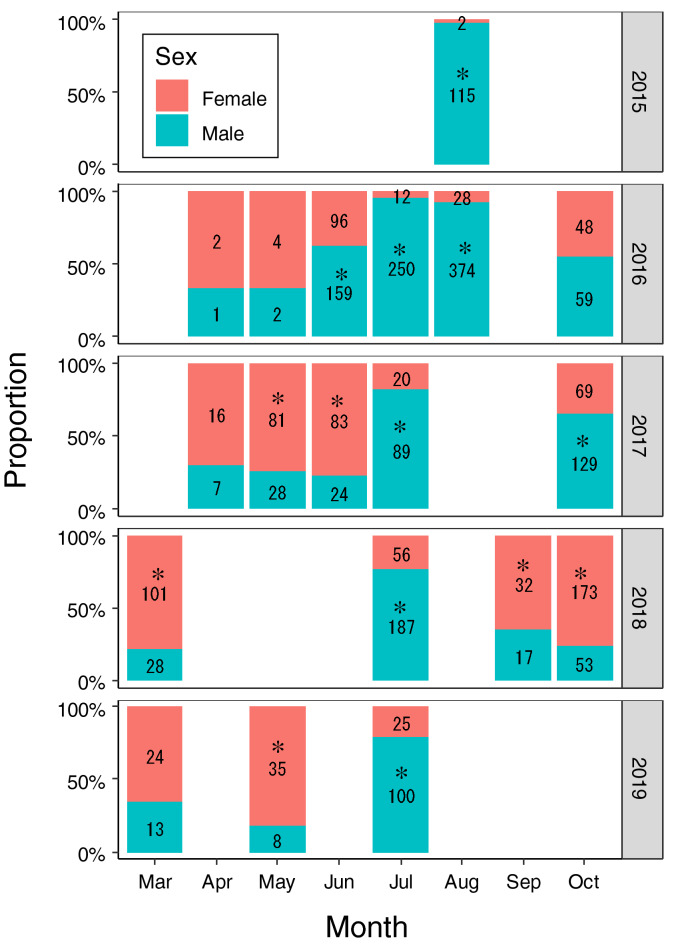
Monthly proportion of females and males of crawling *Laguncula pulchella* individuals (≥ 25 mm shell height) collected in Matsukawaura Lagoon between 2015 and 2019. Numerals show the number of individuals. Asterisks above the numerals indicate the significant difference from 1:1 (*P* < 0.05, binomial test).

Of the 2566 crawling individuals ≥ 25 mm SH that were collected from the lagoon and sexed throughout the study period, females and males comprised 35.9% (922 individuals) and 64.1% (1644 individuals), respectively. The sex ratio varied between months and years (Fig. 4), but it was consistently biased towards males in July and August for each year (binomial test, *P* < 0.001 for all cases). The positive coefficients in July and August in the GLMM (*P* < 0.001, Table 1) indicated a greater proportion of males in these months than in March. During other months, the proportion of females was often greater than that of males (Fig. 4), and a significant difference in the sex ratio from March was observed for May (*P* < 0.01, Table 1). The sex ratio did not differ between day and night (Table 1).

**Table 1:** Summary (coefficient) of the selected generalized linear mixed model (binomial family and logit-link function) for female (0) or male (1) of crawling *Laguncula pulchella* individuals with shell height ≥ 25 mm.

Parameter	Estimate	SE	*P*
(Intercept)	− 0.0053	0.508	0.99
Month (Apr)	− 0.756	0.488	0.12
Month (May)	− 0.897	0.287	0.002
Month (June)	− 0.348	0.260	0.18
Month (July)	2.264	0.213	< 0.001
Month (Aug)	2.070	0.325	< 0.001
Month (Sep)	0.608	0.355	0.087
Month (Oct)	0.261	0.224	0.24

The initial explanatory variables were month and binomial day or night, but the binomial day or night was eliminated from the model based on the Akaike information criterion (AIC). The effect of month was evaluated based on "March".

### Somatic condition

**Figure 5:**
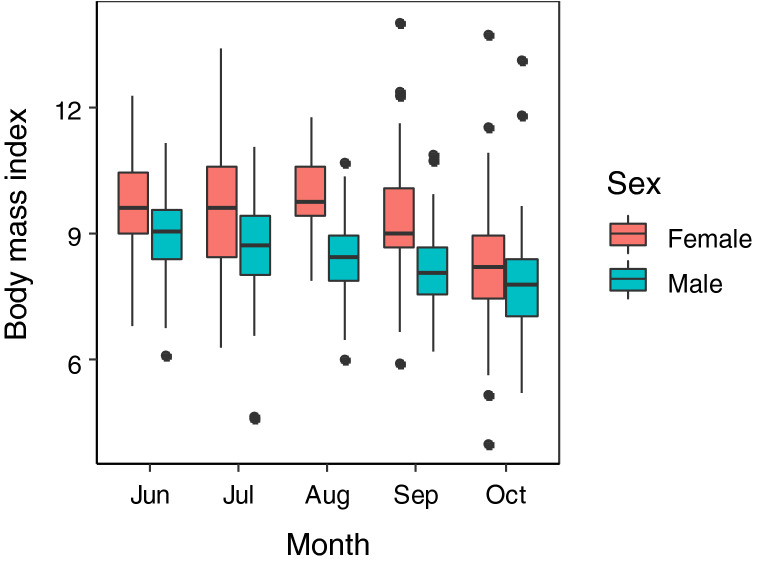
Boxplot of body mass index (BMI) of females and males of *Laguncula pulchella* (≥ 25 mm shell height) collected in Matsukawaura Lagoon between June and October. Data from 2015 to 2018 were pooled (the data each year are shown in ESM Fig. S1). Boxes show the 25% and 75% quartiles and median, vertical bars represent the maximum and minimum values, and solid circles represent outliers.

The body mass index (BMI) was determined for 429 females and 717 males (both with SH ≥ 25 mm). The BMI was consistently lower in males than in females (Fig. 5, ESM Fig. S1, Table 2). The BMI of males gradually decreased from June to October, whereas that of females seemed to be constant from June to September and then markedly decreased from September to October (Fig. 5). The selected GLMM indicated that the BMI of females did not differ between months from June to September, but it was significantly lower in October (Table 2, *P* < 0.001), whereas the BMI of males in August was significantly lower than that in June (*P* < 0.001).

**Table 2:** Summary (coefficient) of the selected generalized linear mixed model (Gaussian family and identity-link function) for body mass index of collected *Laguncula pulchella* individuals with shell height ≥ 25 mm.

Parameter	Estimate	SE	*P*
(Intercept)	8.895	0.369	< 0.001
Shell height	0.018	0.009	0.038
Sex (male)	− 0.664	0.123	< 0.001
Month (July)	0.117	0.205	0.568
Month (Aug)	0.324	0.263	0.219
Month (Sep)	0.339	0.216	0.118
Month (Oct)	− 0.957	0.153	< 0.001
Sex (male): Month (July)	− 0.272	0.227	0.232
Sex (male): Month (Aug)	− 0.944	0.285	< 0.001
Sex (male): Month (Sep)	− 0.370	0.318	0.245
Sex (male): Month (Oct)	0.057	0.184	0.756

Data from June to October were used. All initial explanatory variables were included in the selected model, based on the Akaike information criterion (AIC). The effects of month and sex were evaluated on the basis of "June" and "female", respectively.

## Discussion

The present study is the first to elucidate that the sex ratio of crawling *L. pulchella* individuals was biased towards males from July to August, during which crawling individuals were abundant. We actually observed an individual chasing and mounting another individual in July 2017, and the abundance of mounting pairs also increased from June to July in 2017 and 2019. These findings strongly suggest that males appear on the sand surface to search for mates for copulation. Crawling behavior enables males to successfully encounter a mate. As such, crawling behavior is likely to be for both hunting and mating.

The emergence of naticids on the sediment surface is generally considered a hunting behavior^10,12^. However, if naticid snails only came to the surface for hunting, fewer snails should be observed crawling. Naticid drilling takes a relatively long time; for e.g., *Neverita lewisii* completes its feeding at a rate of ~ 0.1 clams per day^28,29^. A previous field experiment revealed that the *L. pulchella* individual consumes asari clams at a rate of ≤ 0.36 prey per day^18^. Based on the two assumptions that all individuals keep foraging (searching, handling, and consuming prey) and that the emergence of individuals on the sediment surface is necessary to capture prey, we concluded that each individual should come to the sand surface at a frequency of once every 3 days. In other words, the number of crawling individuals should be equivalent to almost one-third of the entire population. However, considering that *L. pulchella* can capture prey and consume it in the substratum^18,24^, the proportion of crawling individuals to the total number of individuals should be small. Actually, the density of crawling individuals (< 0.18 individuals m^−2^, Fig. 2) was very low, compared to that of buried *L. pulchella* individuals (0.4–1.2 individuals m^−2^ sieved by a 9 mm mesh in Matsukawaura Lagoon^15^ or 1.1–13.7 individuals m^−2^ sieved by a 2 mm mesh in Tona coast^19^).

Females may cease to appear on the sand surface during the copulation period. In 2016, the densities of crawling females, as determined by multiplying the proportion of females and density of crawling individuals, were 2.8, 0.4, and 0.3 individuals per 100 m^2^ in June, July, and August, respectively, which were markedly smaller than those of males (3.9, 8.1, and 3.6 individuals, respectively). Females might choose to remain in the sand during July and August, as females can store sperm^17,26^ and the cost of mating is high^30^. However, the feeding activity of females would not be reduced from June to September because of the constant BMI; the decrease in BMI of females in October is probably attributed to egg collar production. Additionally, the sex ratio was not biased towards males for small individuals (both crawling and burrowing) or burrowing mature individuals in July: we collected 14 females and 19 males of crawling small individuals (12–25 mm SH), 17 females and 19 males of buried mature individuals (buried without trails; 27–50 mm SH), and 4 females and 3 males of buried small individuals (16–23 mm SH) in July 2018, and no significant sex bias was observed in any of the cases (*P* > 0.48). This result indicated that only mature males notably increased their crawling activity in July. Another possible explanation for why crawling females were less abundant is that mature females minimize energy loss by remaining in the sand under high aerial temperatures during July and August. Such sex-specific responses to aerial temperature have been observed in another intertidal gastropod *Nucella ostrina*^31^.

It is unclear whether males come to the sand surface only for copulation in summer. Males, like females, might continue their foraging in July and August as we observed that some crawling individuals started capturing prey clams during our field surveys in July and August, although no quantitative evaluation was carried out in the present study. However, if crawling males capture prey and feed on it, it may be disadvantageous from the viewpoint of mating success because it takes around 3 days to consume a clam^18^. Thus, it is expected that males will prioritize mating over foraging from July to August. In general, mating behavior is more energy-consuming than non-mating behavior^32^. The decreasing somatic condition (BMI) from June to August in males supports their reduced foraging activity.

The male-biased sex ratio in crawling individuals was not observed in September and October, but mounting pairs were observed. Although the mechanism of this phenomenon is unclear, active mate searching by males might be concentrated in July and August. The crawling behavior may have a risk of predation. For example, mature males of burrowing amphipods expose themselves to search for females and are susceptible to piscine predation^33,34^. Dogwhelk foraging behavior is also influenced by predation risk at intertidal flats^35^. Although predation on exposed *L. pulchella* individuals has not been recorded, exposing themselves may have a risk of predation by crabs or birds. For instance, predation of *L. pulchella* by the swimming crab *Charybdis (Charybdis) japonica* has been observed in the laboratory^17^. Sex-biased predation risk can alter the growth and production of prey^36^. Thus, energy-consuming mating might become less frequent in September and October, which is the egg-laying season of *L. pulchella*. Another explanation for the relatively high density of exposed individuals in September and October is that both males and females must recover from reduced somatic conditions due to energetic investment in mating from July–August.

This study investigated the daytime crawling behavior of *L. pulchella*. The day or night variable was eliminated from the selected model for the sex of crawling individuals, indicating that the sex ratio of crawling individuals is consistent at any time of the day. However, it is empirically known that crawling individuals are abundant during low tide during the night in autumn and winter^17^; this is possibly related to the lower tidal level during the night than during the daytime. During the copulation period from July to August, the tidal level is relatively high throughout the night, indicating a higher occurrence of crawling individuals during the daytime than during the night. However, it is still unclear why they come to the sand surface during low tide and whether *L. pulchella* appears on the sand surface during the low tide of the neap tide. Future studies may elucidate the detailed biology of naticid crawling behavior such as seasonal variation in the frequency of hunting/mating or crawling frequency under food-limited conditions.

In conclusion, this study is the first to reveal the male-biased sex ratio in crawling naticids during the copulation season. Fishermen should take the following suggestions into consideration for their extermination efforts: (1) crawling *L. pulchella* individuals are abundant, especially in July and August, but they are male-biased; (2) the size distribution of collected *L. pulchella* individuals may be small in July and August, but this may be attributed to the greater proportion of males with smaller size than females; and (3) to reduce the number of females that lay egg collars, constant extermination efforts from May to October, especially during May–June and September–October, should be implemented.

## Methods

### Study site

Field surveys and the collection of *L. pulchella* individuals were conducted in Matsukawaura Lagoon, Fukushima, Japan (37°49′N, 140°59′E) between August 2015 and July 2019. This lagoon has an area of 6 km^2^. The maximum tidal range is approximately 1.5 m. Tidal flats appear during spring low tide, especially during the daytime, from March to September and during the night from October to February. We chose a sandy tidal flat of 30,000 m^2^ in area as the study site (Site F in a previous study^37^). The asari clam *R. philippinarum*, a target species of the clam fishery^38,39^ and the preferred prey of *L. pulchella*^19,40^, densely inhabits this site (average density of 480 individuals m^−2^ in 2015^37^).

### Field survey

Field surveys on crawling *L. pulchella* were conducted once or twice per month during the daytime low tide (0800–1500 h) between March and October from 2015 to 2019. Crawling individuals on the tidal flat were collected by hand and brought to the laboratory within 1 h. The density of crawling individuals was recorded by counting the individuals in an area of 18 m × 3 m, following Yoshida et al.^37^. This procedure was repeated multiple times. We included the individuals that were collected from the trails to calculate the density of crawling individuals. The number of pairs that exhibited shell mounting, which is assumed to be copulating behavior, was also recorded. We also carried out collections of crawling *L. pulchella* individuals for 30 min during low tide without recording the surveyed area. By comparing catch per unit effort (CPUE; the number of individuals per 30 min per person) and the density data for 18 m × 3 m areas on the same day (n = 5), we concluded that a 30-min collection effort was equivalent to the survey area of 1045.8 m^2^. Using this value, the CPUE data were converted into density data. The surveyed area was 432–4487 m^2^. We assumed that our collection of *L. pulchella* hardly affected the subsequent survey data; the population size of adult *L. pulchella* inhabiting the study site was estimated to be 10,200–37,800 individuals, based on the average egg collar density (0.17, 0.63, and 0.30 egg collars m^−2^ in 2015, 2016, and 2018, respectively) and single egg-collar production per female per year^26^.

Additional nocturnal collections of *L. pulchella* individuals were conducted during low tide (2000–2330 h) in March 2018, October 2018, March 2019, and May 2019. Data from these nocturnal surveys were not included in the analysis for density, but for sex ratio (see Measurement and analysis). Collected individuals were brought to the laboratory within 1 h.

### Measurement and analysis

The shell height (SH) of all collected *L. pulchella* was measured to the nearest 0.1 mm using a sliding caliper. The shell was crushed using a hammer, and the soft body of each individual was extracted from the shell using forceps. Soft body wet weight (SBWW, g) was determined to the nearest 0.01 g. The operculum was not included in the soft body. The sex of each individual was identified by gonad inspection (Fig. 1d) or the presence or absence of a penis (Fig. 1e,f).

The densities of crawling *L. pulchella* individuals and mounting pairs were expressed as the number of individuals or pairs per 100 m^2^. To test whether crawling females were larger than crawling males, the SH data of males and females of different months and years were pooled and compared using the Mann–Whitney *U* test and Kolmogorov–Smirnov test.

To test if the sex ratio of crawling *L. pulchella* deviated from 1:1, a binomial test (two-sided) was performed for each month of each year. Individuals assumed to be mature (≥ 25 mm SH^17^) were included in the test. Additionally, to test whether the sex ratio of crawling individuals varied between months, day/night, or both, a generalized linear mixed model (GLMM) with a binomial family and logit-link function was constructed. The response variable was the sex of each individual (female = 0, male = 1), potential explanatory variables were month and day/night, and year was incorporated as a random variable. The model was selected based on the Akaike information criterion (AIC).

To assess the somatic condition of *L. pulchella* individuals, body mass index (BMI) was calculated using the following formula: $\mathrm{BMI}={\text{SBWW}}\times {\mathrm{SH}}^{-2.5}\times {10}^{4}$. The power of 2.5 for SH was derived using a generalized linear model with a Gaussian family and log-link function for SBWW; log (SH), sex, month, and the interaction between sex and month were used as initial explanatory variables. The coefficient of log (SH) was found to be 2.506 in the model selected based on the AIC. To evaluate the effect of sex and month on somatic condition, another GLMM with a Gaussian family and identity-link function was constructed, using BMI as a response variable. The initial explanatory variables were SH, sex, month, and the interaction between sex and month; year was incorporated as a random variable. The model was selected based on AIC.

All statistical analyses were performed using the software R version 4.1.0 (www.r-project.org). Models were constructed using the package “lme4”. The field survey and laboratory procedures were conducted following the guidelines of the Hiroshima University Animal Research Committee (registration number 017A191002) and fishery adjustment regulation of Fukushima Prefecture.

## Supplementary Information

Supplementary Information.

## Data Availability

The datasets generated during and/or analyzed during the current study are available from the corresponding author on reasonable request.
