# Supplementary material for: Male-biased sex ratio in the crawling individuals of an invasive naticid snail during summer: implications for population management
Source: Sci Rep. 2022 May 12;12:7911. doi: 10.1038/s41598-022-12144-1 (PMC9098859; doi:10.1038/s41598-022-12144-1)
Supplement: Supplementary file 1 — Supplementary Information. [file 41598_2022_12144_MOESM1_ESM.pdf]

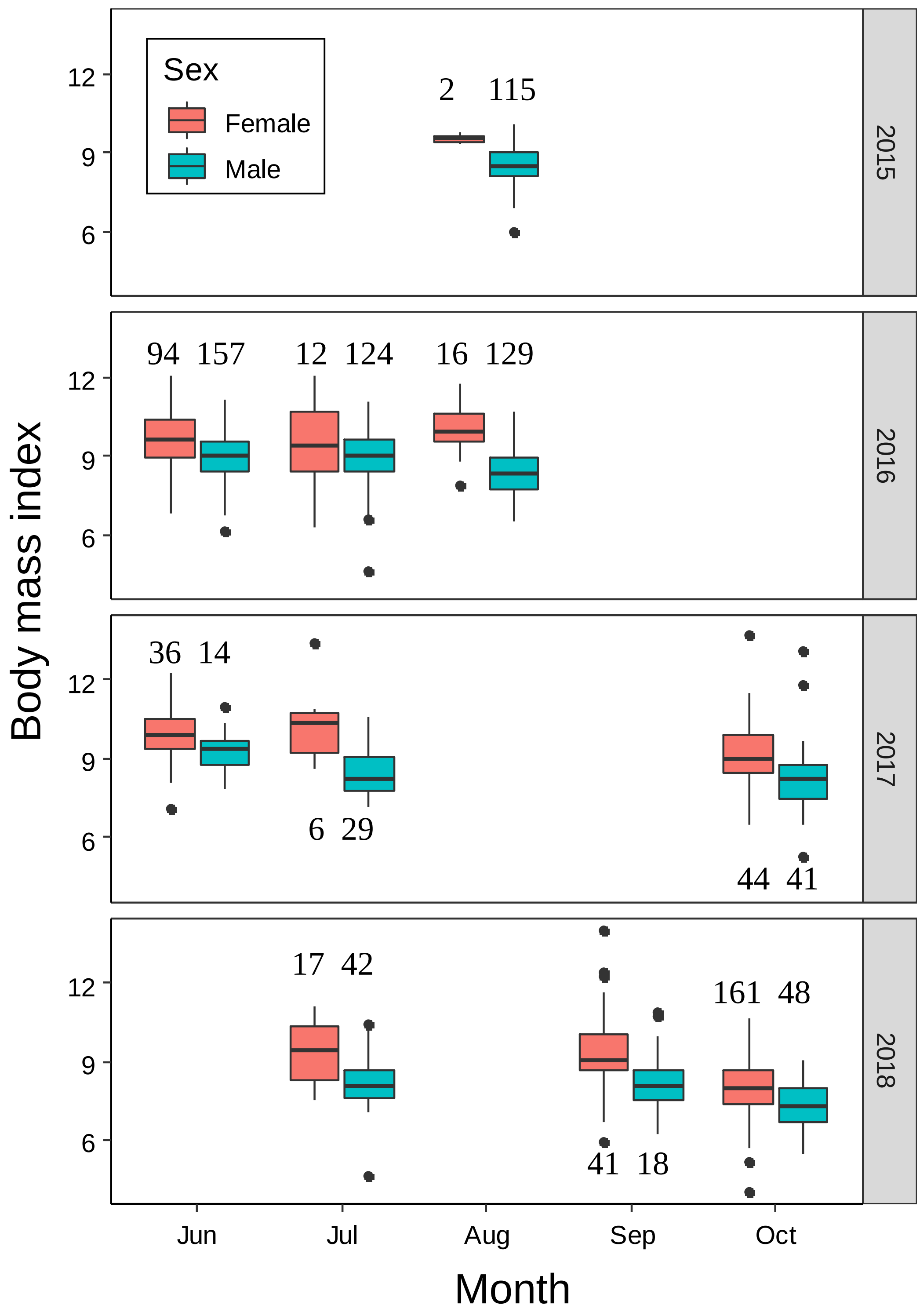

Fig. S1. Boxplot of body mass index (BMI) of females and males of *Laguncula pulchella* (≥25 mm shell height) collected in Matsukawaura Lagoon from June to October in 2015–2018. Boxes show the 25% and 75% quartiles and median, vertical bars show the maximum and minimum values, and solid circles show outliers. Numerals show sample sizes.
